# Evaluation of Synergetic Anticancer Activity of Berberine and Curcumin on Different Models of A549, Hep-G2, MCF-7, Jurkat, and K562 Cell Lines

**DOI:** 10.1155/2015/354614

**Published:** 2015-06-29

**Authors:** Acharya Balakrishna, M. Hemanth Kumar

**Affiliations:** University of Patanjali, Haridwar, Uttarakhand, India

## Abstract

Ayurvedic system of medicine is using *Berberis aristata* and *Curcuma longa* herbs to treat different diseases including cancer. The study was performed to evaluate the synergetic anticancer activity of Berberine and Curcumin by estimating the inhibition of the cell proliferation by cytotoxicity assay using MTT method on specified human cell lines (A549, Hep-G2, MCF-7, Jurkat, and K562). All the cells were harvested from the culture and seeded in the 96-well assay plates at seeding density of 2.0 × 10^4^ cells/well and were incubated for 24 hours. Test items Berberine with Curcumin (1 : 1), Curcumin 95% pure, and Berberine 95% pure were exposed at the concentrations of 1.25, 0.001, and 0.5 mg/mL, respectively, and incubated for a period of 48 hours followed by dispensing MTT solution (5 mg/mL). The cells were incubated at 37 ± 1°C for 4 hours followed by addition of DMSO for dissolving the formazan crystals and absorbance was read at 570 nm. Separate wells were prepared for positive control, controls (only medium with cells), and blank (only medium). The results had proven the synergetic anticancer activity of Berberine with Curcumin inducing cell death greater percentage of >77% when compared to pure curcumin with <54% and pure Berberine with <45% on average on all cell line models.

## 1. Introduction

Cancer is the third leading cause of death worldwide, preceded by cardiovascular and infectious diseases. It is a generic term for a group of more than 100 diseases that can affect any part of the body. Chemotherapy is one of the methods of treating cancer. However the chemotherapeutic drugs are highly toxic and have devastating side effects. Various new strategies are being developed to control and treat several human cancers. Over 60% of anticancer drugs available in the market are of natural origin. Natural products are also the lead molecules for many of the drugs that are in use [1]. Therefore, the phytochemicals present in several herbal products and plants may have the potential to act as preventive or therapeutic agents against various human cancers [2]. Medicinal plants have been in use since time immemorial and their utility has been increasing day by day in the present world. Naturally obtained compounds are considered safer and more easily biodegradable than synthetic compounds and the problem of drug resistance observed in synthetic drugs is also reduced [3]. Plants represent a source of leads for many pharmaceutical compounds and the phytochemical compounds and secondary metabolites present in plants have been used in treating a number of human ailments. Drugs obtained from medicinal plants comprise 25% of total drugs in developed countries and about 80% in developing countries [4].

*Berberis aristata*, commonly known as “Daru haldi,” is a spinous herb native to Northern Himalayan region. This plant is widely distributed from Himalayas to Sri Lanka, Bhutan, and hilly areas of Nepal [5]. It contains mainly Berberine a bitter-tasting, yellow, plant alkaloid with a long history of medicinal use in Chinese and Ayurvedic medicine. Berberine, an isoquinoline alkaloid, belongs to the structural class of protoberberines. It is present in the roots, rhizome, and stem bark of a number of important medicinal plant species including* Hydrastis canadensis* (goldenseal),* Coptis chinensis* (coptis or golden thread),* Berberis aquifolium* (Oregon grape), and* Berberis vulgaris* (barberry). Clinical trials have been conducted using Berberine [6]. There is some evidence to support its use in the treatment of trachomas (eye infections), bacterial diarrhea, and leishmaniasis (parasitic disease). Berberine has also shown antimicrobial activity against bacteria, viruses, fungi, protozoans, helminthes (worms), and chlamydia (STD). Future clinical research is warranted in these areas, as well as cancer, cardiovascular disease, skin disorders, and liver disorders [7].

*Curcuma longa*, a perennial herb and member of the Zingiberaceae (ginger) family, grows to a height of three to five feet and is cultivated extensively in Asia, India, China, and other countries with a tropical climate [8, 9]. Curcumin, a polyphenol with a diarylheptanoid structure that contains two *α*, *β*-unsaturated ketones, is considered to be the major active constituent of turmeric. The chemical properties and the historical background of Curcumin have been reviewed elsewhere [10, 11]. This nontoxic natural compound has been reported to possess several biological activities that are therapeutically beneficial to cancer treatment. Turmeric is used extensively in foods for its flavor and color, as well as having a long tradition of use in the Chinese and Ayurvedic systems of medicine, particularly as an anti-inflammatory, and for the treatment of flatulence, jaundice, menstrual difficulties, hematuria, hemorrhage, and colic [12, 13]. Turmeric can also be applied topically in poultices to relieve pain and inflammation. The active constituents of turmeric are the flavonoid Curcumin. Current research has focused on Curcumin antioxidant, hepatoprotective, anti-inflammatory, anticarcinogenic, and antimicrobial properties, in addition to its use in cardiovascular disease and gastrointestinal disorders [14, 15].

Till date researchers had focused on individual phytochemical derivatives to study anticancer activity and by using individual cancer cell lines. For the first time in our study we have studied combination of two different phytochemicals like Berberine from* Berberis aristata* and Curcumin from* Curcuma longa* on different types of cancer cell line models like A549 (lung cancer cell line) [3], Hep-G2 (liver cancer cell line), MCF-7 (breast cancer cell line), Jurkat (leukemia cancer cell line), and K562 (kidney cancer cell line) which can bring synergetic activity.

## 2. Materials and Methods

### 2.1. Preparation of Berberine and Curcumin

The test items Berberine 95% and Curcumin 95% were purchased from Patanjali Natural Colorama and stored in ambient conditions for further study.

### 2.2. Preparation of Stock Solution

Stock concentration of the test items Berberine and Curcumin and their combination in 1 : 1 ratio were prepared by dissolving the test item in 100% DMSO shown in Table 1 and final stock solution is prepared as shown in Table 2.

### 2.3. Preparation of Positive Control

10% Sodium Lauryl Sulphate (SLS) (w/v) was used as positive control and different concentrations of SLS were used (10, 5, 2.5, 1.25, 0.625, and 0.312 percent solutions).

#### 2.3.1. Preparation of Negative Control

RPMI medium with 0.5% DMSO was taken as negative control.

### 2.4. Description of Cell Lines

All the cell lines described in Table 1 were purchased from National Center for Cell Sciences, Pune, with seeding density of 2.0 × 10^4^ cells/well stored in liquid nitrogen for further testing purpose.

### 2.5. Preparation of MTT Solution

Stock concentration of 5 mg/mL MTT was prepared in PBS and sterile filtered with 0.22 *μ* filter and it was used for the study [3].

### 2.6. Test System Preparation

Prior to the assay the test system A549, Hep-G2, MCF-7, Jurkat, and K562 cells were propagated at 37 ± 1°C in a gaseous environment of 5% ± 1% carbon dioxide, in humid environment in tissue culture flasks containing medium, Dulbecco's Modified Eagle Medium (DMEM) (Invitrogen, USA) supplemented with 10% fetal bovine serum (Invitrogen, USA), and penicillin (100 units) and streptomycin (100 *μ*g) antibiotics (Invitrogen, USA) to obtain the subconfluence of cells (70% to 90% confluent).

#### 2.6.1. Cell Seeding for Cytotoxicity Assessment

Cell monolayer was rinsed with PBS and aspirated off PBS and cells were trypsinized with 0.25% Trypsin with 0.2 g/L EDTA in tissue culture flask at 37 ± 1°C until the cells detached and floated. DMEM with 10% FBS was added into the flask to flush out the cells and centrifuged at 900 rpm for 5 minutes. Cells were resuspended in DMEM medium and cell suspension was subjected for the cell count and viability to determine cell number per mL. Cell number was adjusted to 2 × 10^5^ cells/mL and 0.1 mL of the adjusted cells was seeded in each well of 96-well cell culture plates. Frequent mixing was done during the seeding, to achieve a uniform cell suspension for plating the cells per well. Plates were designated to indicate its contents and date of experiment. Plates were incubated at 37 ± 1°C for 24 ± 1 hrs in gaseous environment of 5% ± 1% carbon dioxide.

After 24 ± 1 hrs of incubation the cells were exposed to different concentrations of test items, Table 3, by replacing the spent medium with 100 *μ*L of different concentrations of the test items solution and incubated for 48 ± 1 hrs at 37 ± 1°C in gaseous environment of 5 ± 1% carbon dioxide. Positive, negative control and blank were dispensed in the designated wells and incubated for 48 ± 1 hrs at 37 ± 1°C in gaseous environment of 5 ± 1% carbon dioxide. At the end of the 48 ± 1 hrs incubation medium with test item/positive control was removed and cells were incubated for 4 hrs with 20 *μ*L of MTT 5 mg/mL solution. After 4 hours of incubation formazan crystals formed by mitochondrial reduction of MTT were solubilized by adding 150 *μ*L of DMSO. Absorbance was read at 570 nm after 10 min incubation with vortexing.

### 2.7. Data Analysis

A decrease in the number of living cells results in a decrease in the metabolic activity in the sample. This decrease directly correlates to the amount of formazan formed as monitored by optical density at 570 nm. Percent viability will be calculated using the following formula [3]:

(1) %Viability=100  O.D  Test  ItemO.D  of  Control,%Activity=100−%Viability.

## 3. Results

Test results and the graphical representation of the study are summarized in Tables 4–7 and represented in Figures 1 [Fig fig2] [Fig fig3] [Fig fig4] [Fig fig5] [Fig fig6] [Fig fig7]–8.

### 3.1. Anticancer Activity on A549 Cells

Pure Berberine and Curcumin were found to have inhibition activity of 64% and 60%, respectively, at a concentration of 0.5 mg/mL and 1.25 mg/mL and combination of Berberine and Curcumin in 1 : 1 ratio was found to bring about 99% inhibition, respectively, at the concentration of 1.25 mg/mL. Positive control SLS showed the 100% inhibition activity.

### 3.2. Anticancer Activity on Hep-G2 Cells

Pure Berberine and Curcumin were found to have inhibitory activity of 85% and 87%, respectively, at a concentration of 0.5 mg/mL and 1.25 mg/mL. Combination of Berberine and Curcumin in 1 : 1 ratio was found to bring about 99% inhibition, respectively, at the concentration of 1.25 mg/mL. Positive control SLS showed the 100% inhibition activity.

### 3.3. Anticancer Activity on MCF-7 Cells

Pure Berberine and Curcumin were found to have inhibitory activity of 87% and 87%, respectively, at a concentration of 0.5 mg/mL and 1.25 mg/mL. Combination of Berberine and Curcumin in 1 : 1 ratio was found to bring about 99% inhibition, respectively, at the concentration of 1.25 mg/mL. Positive control SLS showed the 99% inhibition activity.

### 3.4. Anticancer Activity on Jurkat Cells

Pure Berberine and Curcumin were found to have inhibitory activity of 85% and 23%, respectively, at a concentration of 0.5 mg/mL and 1.25 mg/mL. Combination of Berberine and Curcumin in 1 : 1 ratio was found to bring about 112% inhibition, respectively, at the concentration of 1.25 mg/mL. Positive control SLS showed the 100% inhibition activity.

### 3.5. Anticancer Activity on K562 Cells

Pure Berberine and Curcumin were found to have inhibitory activity of 23% and 164%, respectively, at a concentration of 0.5 mg/mL and 1.25 mg/mL. Combination of Berberine and Curcumin in 1 : 1 ratio was found to bring about 167% inhibition, respectively, at the concentration of 1.25 mg/mL. Positive control SLS showed the 100% inhibition activity.

## 4. Discussion

Ayurvedic system of medicine is using* Berberis aristata* and* Curcuma longa* herbs to treat different diseases including cancer [5]. The study was performed to evaluate the synergetic anticancer activity of Berberine and Curcumin by estimating the inhibition of the cell proliferation by cytotoxicity assay using MTT method [3] on specified human cell lines (A549, Hep-G2, MCF-7, Jurkat, and K562). All the cells were harvested from the culture and seeded in the 96-well assay plates at seeding density of 2.0 × 10^4^ cells/well and were incubated for 24 hours. Test items Berberine with Curcumin (1 : 1), Curcumin 95% pure, and Berberine 95% pure were exposed at the concentrations of 1.25, 0.001, and 0.5 mg/mL, respectively, and incubated for a period of 48 hours followed by dispensing MTT solution (5 mg/mL). The cells were incubated at 37 ± 1°C for 4 hours followed by addition of DMSO for dissolving the formazan crystals and absorbance was read at 570 nm. Separate wells were prepared for positive control, controls (only medium with cells), and blank (only medium). The exhibited IC-50 values of combination of Berberine and Curcumin were estimated to be 0.034, 0.047, 0.022, 0.2, and 0.01 mg/mL in Hep-G2, A549, MCF-7, Jurkat, and K562 cells, respectively. The exhibited IC-50 values of pure Curcumin were estimated to be 0.020, 0.014, and 0.011 mg/mL in Hep-G2, A549, and MCF-7 cells, respectively and the IC-50 calculation was not feasible in both the cells Jurkat and K562. The exhibited IC-50 values of pure Berberine were estimated to be 0.20 and 0.1 mg/mL in Hep-G2 and A549 but, in MCF-7 IC-50, Jurkat and K562 were unable to be estimated. The in vitro screenings of Berberine and Curcumin combination were found to be having an inhibiting activity against all the three cell lines. Percent inhibition activity at the concentrations 1.25, 0.313, and 0.078 mg/mL was estimated to be greater than 77% in A549, Hep-G2, and MCF-7 cells. The in vitro screenings of Berberine and Curcumin combination were found to be having an inhibiting activity against K562 cells. Percent inhibition activity at the concentrations 1.25, 0.313, and 0.078 mg/mL was estimated to be greater than 68% in K562 cells. The results had proven the synergetic anticancer activity of Berberine with Curcumin inducing cell death greater percentage of >77% when compared to pure Curcumin with <54% and pure Berberine with <45% on average on all cell line models.

## 5. Conclusion

In conclusion, we confirmed that the combination of Curcumin and Berberine synergistically generates anticancer effects in A549, Hep-G2, MCF-7, Jurkat, and K562 cells in vitro, possibly mediated by inducing apoptosis. With regard to A549, Hep-G2, MCF-7, Jurkat, and K562 Curcurmin and Berberine are of extreme antitumor agents. The combination of Curcumin and Berberine is a novel strategy that has potential in the treatment of cancer patients.

## Figures and Tables

**Figure 1:**
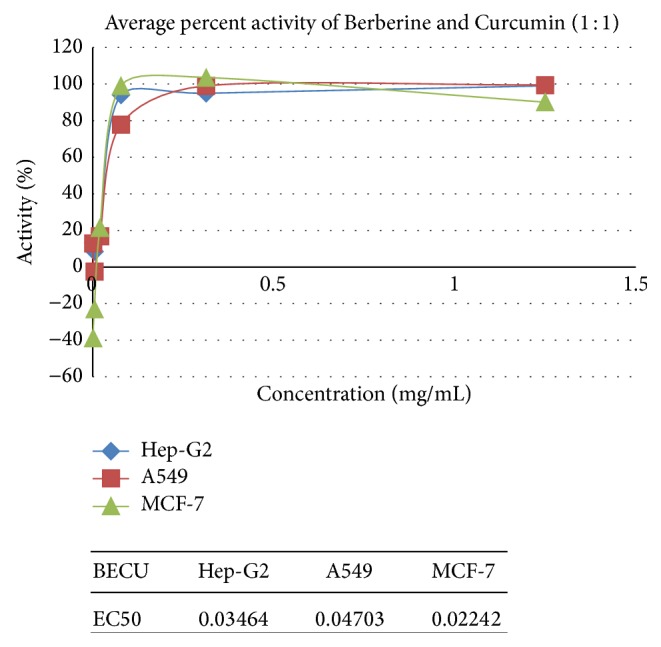
Synergetic anticancer activity of Berberine and Curcumin on A549, Hep-G2, and MCF-7.

**Figure 2 fig2:**
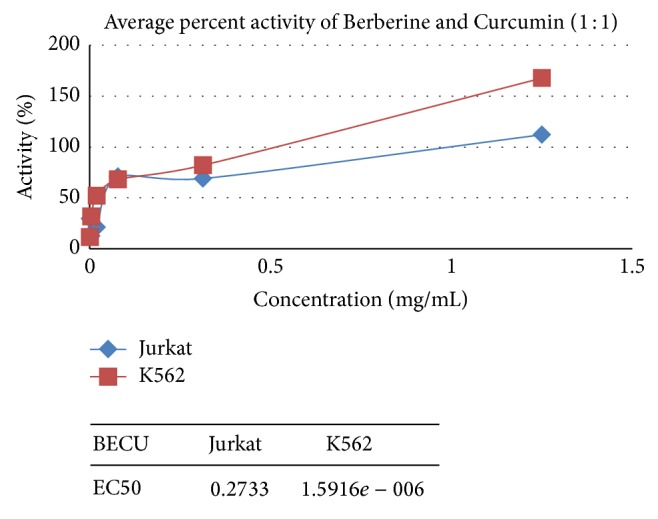
Synergetic anticancer activity of Berberine and Curcumin on Jurkat and K562.

**Figure 3 fig3:**
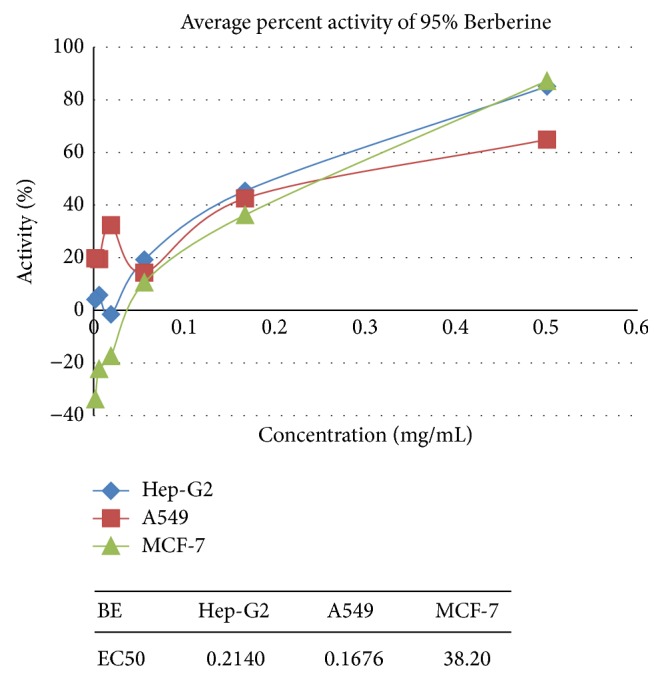
Anticancer activity of Berberine on A549, Hep-G2, and MCF-7.

**Figure 4 fig4:**
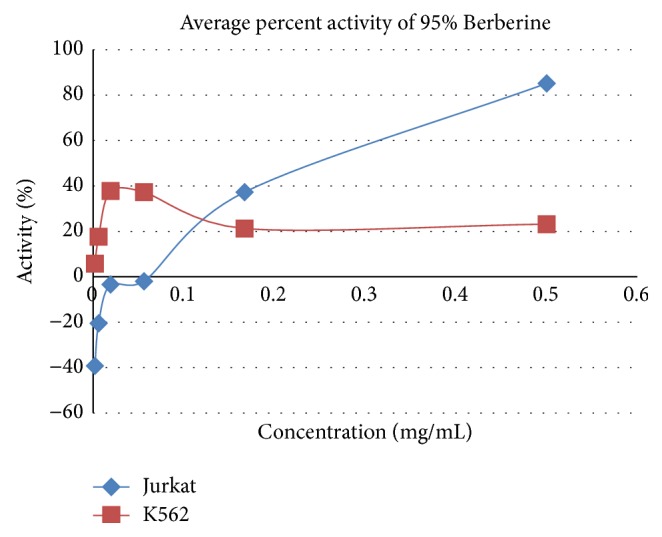
Anticancer activity of Berberine on Jurkat and K562.

**Figure 5 fig5:**
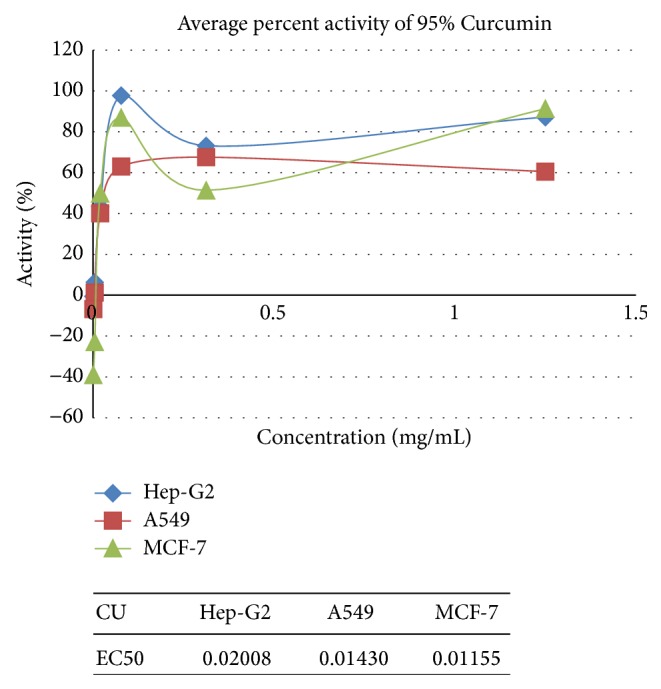
Anticancer activity of Curcumin on A549, Hep-G2, and MCF-7.

**Figure 6 fig6:**
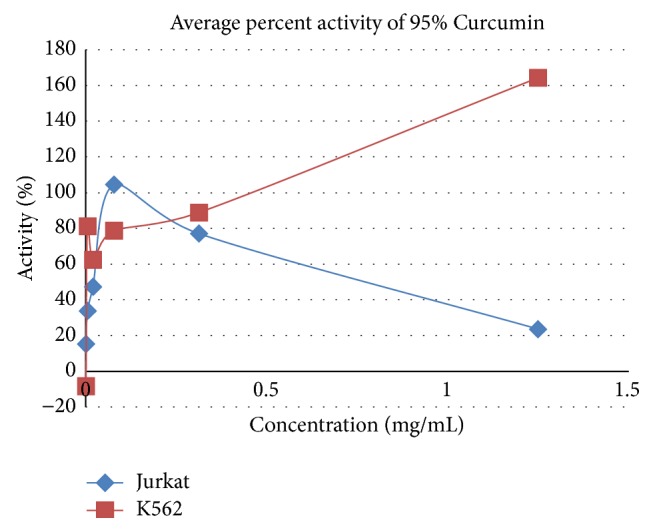
Anticancer activity of Curcumin on Jurkat and K562.

**Figure 7 fig7:**
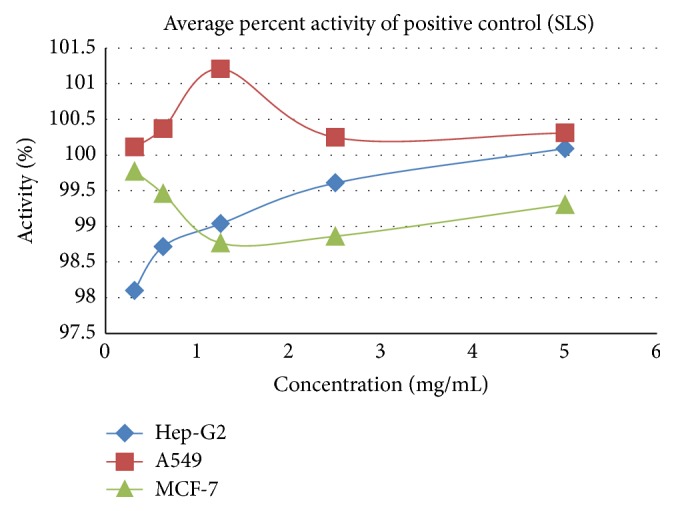
Cytotoxicity effect of SLS positive control on A549, Hep-G2, and MCF-7.

**Figure 8:**
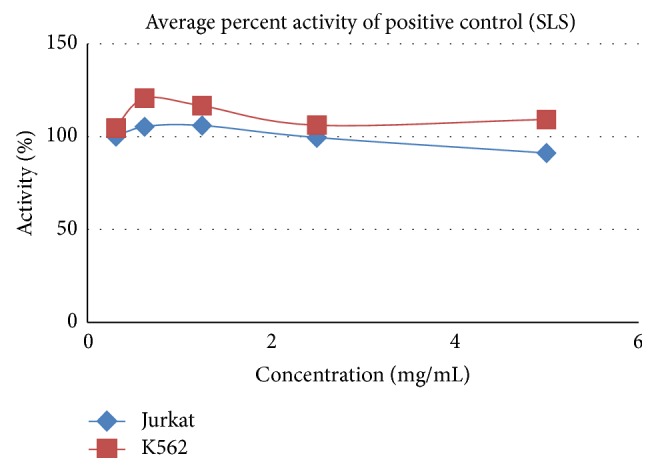
Cytotoxicity effect of SLS positive control on Jurkat and K562.

**Table 1:** Test system description.

S. number	Cell line	Description of cell line	Type of cells
1	Hep-G2	Liver cancer cell lines	Adherent cells
2	MCF-7	Breast cancer cell lines	Adherent cells
3	A549	Lung cancer cell lines	Adherent cells
4	K562	Bone cancer cells	Suspension cells
5	Jurkat	Leukemia	Suspension cells

**Table 2:** Preparation of initial stock concentration of test items.

S. number	Test item name	Initial stock concentration in mg/mL (highest soluble conc. in DMSO)	Volume of DMSO initial stock in mL	Volume of DMEM medium in mL	Final stock in DMEM in mg/mL (1st concentration)
1	Berberine + Curcumin (BECU)	250	0.025	4.975	1.25

2	Curcumin (CU)	250	0.025	4.975	1.25

3	Berberine (BE)	100	0.025	4.975	0.5

From the final stock different concentrations of the final working stocks five concentrations were prepared in DMEM medium by 4-fold and 3-fold serial dilutions as specified in Table 3. Diluted stocks were used for the study.

**Table 3:** Final stock concentrations used in the study.

S. number	Test item name	Final stock in DMEM in mg/mL	Final working stock concentration in RPMI in mg/mL	Volume of working stock in mL	Volume of DMEM in mL
1	Berberine + Curcumin (BECU)	1.25 (4-fold)	0.313, 0.078, 0.020, 0.005, 0.001	1.25 mL	3.75 mL

2	Curcumin (CU)	1.25 (4-fold)	0.313, 0.078, 0.020, 0.005, 0.001	1.25 mL	3.75 mL

3	(Berberine) BE	0.5 (3-fold)	0.16, 0.0565, 0.019, 0.006, 0.002	1.67 mL	3.33 mL

**Table 4:** Average percent activity of Berberine and Curcumin (1 : 1).

Concentration in mg/mL	Average percent activity of Berberine and Curcumin (1 : 1)				
	Hep-G2	A549	MCF-7	Jurkat	K562
1.250	99.055	99.467	90.110	112.080	167.700
0.313	94.986	99.086	103.589	69.003	82.041
0.078	94.110	77.913	99.021	71.054	68.217
0.020	18.959	16.781	21.411	21.026	51.938
0.005	8.548	−2.513	−23.083	12.650	31.783
0.001	13.260	12.947	−38.948	29.573	11.370

**Table 5 tab5:** Average percent activity of 95% Curcumin.

Concentration in mg/mL	Average percent activity of 95% Curcumin				
	Hep-G2	A549	MCF-7	Jurkat	K562
1.250	87.281	60.572	91.339	23.566	164.271
0.313	73.164	67.627	51.388	77.057	88.809
0.078	97.774	63.016	87.008	104.489	78.747
0.020	43.297	40.120	49.814	47.257	62.320
0.005	6.348	1.291	−22.897	33.915	81.211
0.001	−0.474	−6.756	−39.039	15.337	−8.316

**Table 6 tab6:** Average percent activity of 95% Berberine.

Concentration in mg/mL	Average percent activity of 95% Berberine				
	Hep-G2	A549	MCF-7	Jurkat	K562
0.500	85.085	64.878	87.214	85.085	23.130
0.167	45.384	42.561	36.227	37.247	21.188
0.056	19.290	14.390	10.655	−2.023	37.236
0.019	−1.547	32.398	−17.403	−3.372	37.750
0.006	5.816	19.472	−22.336	−20.519	17.533
0.002	4.136	19.797	−33.741	−39.348	5.768

**Table 7:** Average percent activity of positive control (SLS).

Concentration in percentage	Average percent activity of positive control (SLS)				
	Hep-G2	A549	MCF-7	Jurkat	K562
5.000	100.092	100.309	99.305	91.243	109.321
2.500	99.610	100.247	98.861	99.553	106.180
1.250	99.037	101.210	98.764	106.009	116.717
0.625	98.716	100.370	99.459	105.433	120.770
0.313	98.097	100.111	99.768	100.000	104.762
